# A new analogy for the effects of ambient particles on health

**DOI:** 10.1186/1743-8977-10-30

**Published:** 2013-07-16

**Authors:** Robert L Maynard

**Affiliations:** 1 Honorary Professor, Birmingham University, Birmingham, UK

## 

12th July 2013

It is accepted that exposure to the ambient aerosol has a wide range of effects on health. Effects are related both to long-term exposure and to short-term exposure and include effects on the cardiovascular and respiratory systems, on prematurity of birth and retardation of intra-uterine development and on the central nervous system. Such a range of effects of exposure to low concentrations of particles of varying size and composition has been difficult to explain. I believe that this difficulty has been compounded by two strands of thinking, neither of which is appropriate.

These strands of thinking have been based on two well established principles: that of physiological homeostasis and that of the specificity of effects of individual toxicologically active substances. The former has led to difficulties in accepting that exposure to low concentrations of compounds found in the ambient aerosol could disturb physiological functioning; the latter has led to the assumption that specific components of the aerosol are responsible for specific effects on health. The error as regards the former is to assume that normal homeostatic processes apply to disease states; the error in the latter is to think at a molecular rather than at a systems level.

I suggest a new analogy: that of tall columns and squat cones. If we consider a tall column standing on its base it is obvious that anything that adds to the height of the column will move its centre of gravity upwards and will increase its instability; that is its incapacity to resist horizontal displacement of its upper end. A squat cone, on the other hand, has a large capacity to resist being disturbed by horizontal displacement of its point: its centre of gravity is low. Normal physiological systems can be represented by the cone: homeostatic processes resist displacement and stability characterises the systems. Patho-physiological states are better represented by the tall column: as the column becomes taller the force needed to topple it becomes less. Anything that adds to the height of the column increases its instability; anything that increases forces applied horizontally to its upper end increases the likelihood that it will topple.

The wall of an airway in a patient suffering from asthma can be represented by a tall column. Such an airway is described by clinicians as unstable: indeed it is, a wide range of minor, often non-specific, insults can cause it to react and increase airway resistance. The height of the column can be reduced by therapy, for example by anti-inflammatory drugs. An atherosclerotic plaque in a coronary artery can also be represented by a tall column: it is unstable and its instability increases as it develops.

Such thinking dispenses with the misleading concept of an individual agent being critical to an effect of the ambient aerosol. Thus the question of whether nickel, copper, iron, vanadium, zinc, cobalt, PAH compounds or ultrafine particles are essential to the effects of the ambient aerosol, to a large extent, disappears. Anything capable of adding to the height of the column or exerting a rocking force could be playing a part in increasing instability. Whether one factor will have a greater effect than another will, of course, depend on their relative concentrations and relative activities. In one location nickel might be important; in another, perhaps, PAH compounds which contribute to oxidative free radicals might play a more important role. But no one compound, perhaps no one group of compounds, should be regarded as essential to the increase in instability. All that is required is that the aerosol contains materials that can add to instability.

Here a difficulty seems to appear. Need all the compounds act via the same mechanism to increase instability? I can see no requirement for this. As regards plaques, one compound might act by increasing the deposition of a specific type of lipid material in a plaque, another by increasing the influx of inflammatory cells into the plaque, a third by triggering clotting on the disturbed surface of the plaque. Each leads to an increase in instability either by, as it were, increasing the height of the column or by applying horizontal force to its upper end. The key to the effect is the increase in instability and not the specific mechanisms, and there may be many, by which this is produced.

The analogy of an unstable column seems, to me, also to be helpful in explaining how exposure to low concentrations of pollutants can exert an effect. If we imagine the column rocking dangerously close to its tipping point it seems clear that addition of only minimal force could cause it to topple. Thus application of force which would have no effect on column not already rocking, let alone on a squat cone, might be enough to cause a catastrophic effect. To say that the force was sufficient to push the column over is nonsense when looked at in one way but perfectly good sense when looked at in another.

The toppling column seems a good analogy for the effects of pollutants which bring forward the date of a person’s death but at first glance seems less apt when thinking of the development of a disease state. But this might not be so: if we imagine that as disease is induced there is a conversion from a squat cone to a column and then to a taller column we can see that anything which adds height to the column will increase instability. As far as I can see there is no reason to think that pollutants, in addition to diseases, should not have such an effect. One might think of a conflict between a disease process and normal homeostatic mechanisms. For some time the disease process cannot get going, so to speak, because it is resisted by homeostatic processes but as it develops it brings the cone towards a column and then increases the height of the column. We do not know enough about the nature of this process: it may be that it does not flow linearly but in sudden steps or jumps: the jumps being produced when a retarding force (homeostatic force) is overwhelmed. That pollutants could play a role in this seems at least possible. Exposure to the pollutant need not have “caused” the disease, though it might have contributed, but it might well have caused the disease to advance more rapidly past what might be regarded as a series of homeostatic obstructions.

Focusing on mechanisms of effect of individual components of the ambient aerosol is likely to be unhelpful. What needs to be explored is the stability or instability of systems. The columns and cones we have been talking about should be seen as systems: the airway in the patient suffering from asthma, the plaque in a coronary artery, the placental barrier, the neuron-glia unit are all systems and are all, to a greater or lesser extent, afflicted by instability. For most of everybody’s life they are stable (squat cones) but they can become unstable (tall columns): if we could understand the processes underlying this shift in state we might be better able to understand the effects of the ambient aerosol, indeed of air pollutants in general. It is likely that we would be less surprised by the effects of low concentrations of pollutants or by the fact that a wide variety of components of the ambient aerosol appear to be having an effect.

